# Common Biomarkers in Chronic Obstructive Pulmonary Disease and Bronchopulmonary Dysplasia: A Narrative Review of an Intriguing Interplay

**DOI:** 10.3390/ijms27031422

**Published:** 2026-01-30

**Authors:** Antonella Gambadauro, Federica Xerra, Valeria Chirico, Immacolata Rulli, Annalisa Cacciola, Raffaella Mallamace, Eloisa Gitto, Lucia Marina Marseglia

**Affiliations:** 1 Neonatal and Pediatric Intensive Care Unit, Department of Human Pathology in Adult and Developmental Age “Gaetano Barresi”, University of Messina, 98124 Messina, Italy; gambadauroa92@gmail.com (A.G.); federicaxerra@gmail.com (F.X.); rulli.imma74@gmail.com (I.R.); annalisa.cacciola@polime.it (A.C.); raffaella.mallamace@polime.it (R.M.); eloisa.gitto@unime.it (E.G.); 2 Pediatric Nephrology and Dialysis Unit, Department of Human Pathology in Adult and Developmental Age “Gaetano Barresi”, University of Messina, 98124 Messina, Italy; valeriachirico@hotmail.it

**Keywords:** biomarker, BPD, bronchopulmonary dysplasia, infants, intensive care unit, chronic obstructive pulmonary disease, COPD

## Abstract

Bronchopulmonary dysplasia (BPD) is a chronic lung condition in preterm infants characterized by impaired alveolar development, disrupted vascular growth, and persistent inflammation. These alterations, which often arise from early exposure to mechanical ventilation, oxygen toxicity, and infection, can lead to long-term structural and functional deficits in the developing lung. In adulthood, chronic obstructive pulmonary disease (COPD) represents a major cause of morbidity and mortality and is defined by progressive airflow obstruction, reduced respiratory capacity, and chronic inflammatory responses. Although traditionally considered a disease of adult smokers, growing evidence suggests that early-life respiratory insults play a key role in shaping long-term lung health. Recent studies reveal a biologically plausible link between BPD and later COPD, indicating that premature birth, impaired lung growth, and early inflammatory injury may predispose individuals to earlier or more severe COPD development. This review explores the shared molecular pathways connecting these conditions, focusing on overlapping inflammatory biomarkers such as *IL1B*, *IL6*, *IL8*, *TNF*, *TGFB*, and *VEGF*, which collectively reflect persistent dysregulation of immune and repair mechanisms. Additionally, common genetic variants, including *SERPINA1* and *HHIP*, may contribute to susceptibility across the lifespan. Emerging biomarkers—such as *PRMT7*, *cathelicidin*/*LL-37*, *CRISPLD2*, and *GDF15*—offer further insight into disease progression. Identifying these shared markers may ultimately improve early detection and help clinicians pinpoint infants with BPD who face an elevated risk of developing COPD later in life.

## 1. Introduction

Bronchopulmonary dysplasia (BPD) is the most common respiratory disease in infants born prematurely, affecting between 50% to 66% of newborns with birth weight under 1000 g in different epidemiological studies [1]. In the past few years, BPD was associated with aggressive mechanical ventilation and hyperoxygenation, which increased the risk of airway damage, smooth muscle hypertrophy, lung remodeling, neutrophilic inflammation, and parenchymal fibrosis (old form of BPD) [2]. The improvement in the management of preterm infants modified the history of the disease, creating a new form of BPD secondary to a global alveolar development arrest [3]. The new form of BPD is characterized by a significant loss of the surface area for gas exchange, associated with airway damage, inflammation, and fibrosis, which are usually milder than in the old form of BPD [2]. This condition is commonly defined as an oxygen requirement for ≥28 days of postnatal age or 36 weeks of postmenstrual age [3]. BPD in childhood increases the risk of long-term lung impairment [4]. Specifically, affected children often experience a faster decline in lung function and a greater probability of developing respiratory diseases in adulthood [5].

Chronic obstructive pulmonary disease (COPD) is a leading cause of death and morbidity in adults worldwide [6]. COPD is a small airway disease characterized by fixed airway obstruction and an accelerated decline of the predicted forced expiratory volume in the first 1 s (FEV1%) [7]. An increasing number of studies have explored the possible association between early-life lung impairment and the development of chronic obstructive lung disease later in life. Barker et al. reported that men born with low birth weight more frequently exhibit reduced lung function and increased mortality from COPD in adulthood [8]. Although low birth weight is not specific to BPD, it is commonly regarded as a marker of impaired early lung development and increased vulnerability to respiratory disease.

Premature infants with BPD may also develop a disproportion between airway caliber and lung size, a condition known as airway dysanapsis [9]. Dysanapsis has been associated with airflow limitation and has been proposed as a risk factor for adult-onset COPD [8]. However, direct longitudinal evidence linking BPD to the subsequent development of COPD remains limited.

Identifying factors that influence respiratory trajectories in individuals with a history of BPD and clarifying their long-term risk of developing chronic obstructive lung disease represent important goals for future research and may have relevant implications for long-term disease monitoring and management.

The purpose of this narrative review was to analyze biomarkers that have been reported in association with both BPD and COPD. Identifying shared biomarkers may help to identify infants with BPD who are at increased risk of developing COPD later in life.

A computerized literature search was conducted primarily in PubMed using combinations of the following key terms: “biomarker”, “bronchopulmonary dysplasia” or “BPD”, and “chronic obstructive pulmonary disease” or “COPD”. The search was intended to identify relevant and representative studies rather than to provide an exhaustive or systematic assessment of the literature.

Articles were selected based on their relevance to the topic following a manual screening of titles and abstracts. The review was based on a critical qualitative evaluation of the literature and did not follow a standardized systematic methodology or include statistical meta-analyses.

## 2. Pathophysiology and Common Pathways of BDP and CODP

Respiratory health follows a defined lung function trajectory, with maximal lung function reached in early adulthood, followed by a progressive decline with aging [10]. Lung development continues during the postnatal period, which represents a critical window for alveolar growth [11]. Alveolarization predominantly occurs within the first years of life, reaching approximately 300–800 million alveoli by around 8 years of age, after which alveolar number stabilizes while alveolar size continues to increase until adolescence [11]. Disruptions of this finely regulated developmental process may result in a permanently altered lung structure, predisposing individuals to chronic respiratory diseases later in life. In this framework, bronchopulmonary dysplasia (BPD) represents an early-life disease of disrupted lung development, whereas chronic obstructive pulmonary disease (COPD) is a disorder of progressive lung injury occurring later in life; nevertheless, both conditions share several pathogenic mechanisms.

Life-course convergence of early-life and adult risk factors in bronchopulmonary dysplasia (BPD) and chronic obstructive pulmonary disease (COPD). Distinct exposures across different life stages converge on shared inflammatory and remodeling pathways, including persistent low-grade inflammation, cytokine dysregulation, and abnormal lung remodeling, potentially contributing to overlapping structural and functional lung abnormalities.

### 2.1. Early-Life Disruption of Lung Development: BPD

**Figure 1:**
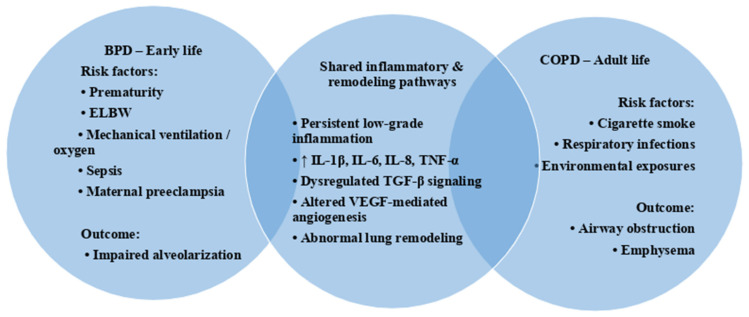
Life-course convergence of inflammatory pathways in BPD and COPD.

Several factors can negatively affect respiratory trajectories and induce an early decline in lung function. Premature birth and the development of BPD represent a major cause of such impairment. Maternal preeclampsia, prematurity, extremely low birth weight, surgical closure of patent ductus arteriosus, sepsis, lung congestion, and barotrauma related to mechanical ventilation all increase susceptibility to BPD (Figure 1) [12]. The contemporary form of BPD is characterized by early interruption of alveolar growth, leading to alveolar simplification, with fewer and larger alveoli, reduced septation, and disrupted pulmonary vascular development [13]. Compared to the historical form of BPD, inflammation and fibroproliferative changes are less pronounced, although they remain common features [13]. Overall, alveolar and vascular simplification represent the main pathological hallmarks of BPD lungs [14].

### 2.2. Inflammatory Pathways in BPD and Long-Term Consequences

Both prenatal and postnatal factors can increase the risk of inflammation and BPD development in neonates. Hyperoxia, hypoxia, endotoxins, bacterial products, and mechanical stress activate neutrophils, alveolar macrophages, fibroblasts, type II pneumocytes, and endothelial cells, leading to the release of multiple pro-inflammatory cytokines [15]. Compared to healthy controls, preterm infants who develop BPD exhibit increased levels of pro-inflammatory cytokines—particularly interleukin (IL)-1β, IL-6, and tumor necrosis factor (*TNF*)—in serum and tracheal aspirates, along with reduced concentrations of anti-inflammatory cytokines such as *IL-4*, *IL-10*, and transforming growth factor (TGF)-β1 [16]. This imbalance contributes to airway injury and disease development (Figure 1). IL-1β plays a pivotal role as a mediator of chronic inflammation and airway remodeling [17]. It promotes lung fibrosis by inducing collagen production from fibroblasts and epithelial cells and stimulates the secretion of glycosaminoglycans and fibronectin [17]. Additionally, IL-1β alters the expression of proteins involved in the retinoic acid signaling pathway, which is crucial for normal lung development [18]. Elevated IL-8 levels have also been observed in tracheal aspirates of infants who develop BPD during the first days of life [19], and experimental exposure of preterm lung explants to hyperoxia results in higher IL-8 concentrations compared to term lungs [16]. TNF exerts pleiotropic effects on inflammatory responses through activation of the nuclear factor kappa-B (NFκB) signaling pathway [20]. In experimental rat models, *TNF* overexpression induces severe pulmonary inflammation, interstitial fibrosis, increased myofibroblast numbers, and enhanced TGF-β production [21]. Clinically, elevated *TNF* levels are associated with chronic inflammation in neonates with BPD, and *TNF* inhibition has been shown to reduce disease risk and severity [22].

### 2.3. Vascular Development and VEGF Signaling in BPD

Vascular endothelial growth factor (*VEGF*) is a key regulator of vasculogenesis, and early alterations in its expression are associated with defects in cardiovascular and pulmonary development [23]. Embryos lacking VEGF receptor 2 (VEGFR2) fail to develop functional blood vessels and do not survive after birth [24]. *VEGF* expression is stimulated primarily by hypoxia via hypoxia-inducible factor 1 (HIF-1) and by pro-inflammatory cytokines such as IL-1β, *TGF-β*, and *TNF*, while VEGFR1 and VEGFR2 expression is also regulated by hypoxic conditions [23,24]. Preterm infants who develop BPD show reduced VEGF levels in tracheal aspirates during the early postnatal period [25], and decreased serum VEGF concentrations have been detected during the first week of life [26]. Early disruption of VEGFR signaling in the neonatal lung may worsen BPD sequelae and increase susceptibility to adult respiratory diseases, including COPD [27].

### 2.4. Chronic Inflammation and Remodeling in COPD

COPD is characterized by chronic inflammation predominantly affecting the lung parenchyma and peripheral airways, leading to progressive airway remodeling and persistent airflow obstruction [28]. This inflammatory process underlies the heterogeneous phenotypes of COPD, including emphysema and chronic bronchitis, the latter primarily involving the small airways [29]. Acute exacerbations are common and are typically associated with increased inflammatory activity triggered by infections and environmental exposures [29,30]. Among environmental risk factors, cigarette smoking plays a central role in COPD development (Figure 1) [31].

Inflammation in COPD involves both innate and adaptive immune responses as well as structural lung cells, including airway and alveolar epithelial cells, endothelial cells, and fibroblasts [28]. Exposure to tobacco smoke and other inhaled irritants stimulates epithelial cells to release inflammatory mediators such as *TNF*, *IL-1β*, *IL-6*, and *IL-8* (Figure 1) [32]. TNF and IL-8 act as potent chemoattractants for macrophages and neutrophils, which represent the predominant inflammatory cells in COPD lungs [33]. Elevated levels of IL-6, IL-8, and TNF have been detected in serum and bronchoalveolar lavage fluid of COPD patients compared to healthy controls, with higher concentrations observed in severe disease [32,33,34]. *TGF-β* is produced by epithelial cells in the small airways of COPD patients and is associated with the development of local fibrosis [28]. Increased TGF-β1 expression has been observed in epithelial cells, macrophages, and fibroblasts isolated from COPD lungs, supporting its role in disease progression [35]. Finally, *VEGF* is essential for maintaining alveolar cell integrity, and experimental inhibition of *VEGF* receptors in animal models induces alveolar cell apoptosis and emphysema-like changes [36].

## 3. COPD and BPD: Common Biomarkers

### 3.1. Genetic Factors

Genetic factors have been studied in animal and human studies as possible key components in the susceptibility to develop BPD and/or COPD (Table 1).

A study on non-Hispanic White and African American individuals indicated a COPD heritability of approximately 37.7% [83]. Twin studies have suggested that genetic susceptibility affects 53–82% of the risk of BPD development [84]. Genome-wide association studies (GWAS) have identified single-nucleotide polymorphisms (SNPs) associated with COPD. Variants in the *SERPINA1* gene are responsible for alpha-1 antitrypsin (A1AT) deficiency and can also be linked to early-onset COPD [85]. Qiu et al. demonstrated a significant association between *SERPINA1* hypomethylation (cg02181506 and cg24621042 sites) and COPD, especially in smokers [86]. A study on baboons revealed low levels of A1AT elastase inhibitory activity in the airways of animals with severe BPD [87]. However, a clear relationship between A1AT deficiency and BPD has not yet been confirmed.

A particular BPD phenotype is pulmonary hypertension (PH) associated with BPD (BPD-PH). This condition is characterized by impaired alveolar diffusion, abnormal pulmonary vascular remodeling, and vascular growth arrest, leading to increased pulmonary vascular resistance and PH [88]. A multiomic approach revealed the involvement of specific pathways in patients with BPD-PH, including *NFKB1*, *VEGFA*, *SERPINA1*, insulin signaling, LDL metabolism, extracellular signal-regulated kinase (MAPK1/2), and IL6 [89]. In previous human studies, overexpression of the NFKB1 gene [90] and the presence of specific VEGFA variants (rs833068G > A, rs833070T > C, and rs3024997G > A) were associated with an increased risk of COPD development [39]. Among microRNAs (miRNAs), miR-206 downregulation and upregulation were associated with BPD and COPD, respectively [40]. However, future studies are needed to determine whether these genetic variants may predict COPD susceptibility in infants with BPD.

COPD-associated gene variants are more frequently found in children with BPD, particularly in severe cases. Studies in mice have suggested an association between specific genetic variants and COPD-like phenotypes, especially in the presence of tobacco smoke exposure [38]. A study in the human population reported that HHIP variants were associated with a high risk of COPD development [91]. HHIP is part of the Hedgehog signaling pathway, which plays a pivotal role in early lung development and responses to injury [37,91]. An observational cohort study including extremely low-birth-weight infants showed that children who subsequently developed BPD had higher early airway HHIP protein levels compared to those without BPD [41]. Moreover, this study reported that the HHIP variant rs13147758 (GG genotype) was independently protective against BPD onset [41]. Future meta-analyses and GWAS could help identify SNPs causally linked to BPD and COPD.

### 3.2. Inflammatory Biomarkers

COPD and BPD are characterized by chronic inflammation, which leads to modifications in lung function and morphometry. Common inflammatory markers in BPD and COPD may help establish a link between these two conditions and aid in identifying BPD infants at higher risk of developing COPD later in life.

#### 3.2.1. IL-1β, IL-6, and IL-8

Elevated levels of pro-inflammatory ILs, such as IL-1β, IL-6, and IL-8, have been observed in patients with COPD and BPD. Circulating IL-1β, IL-6, and IL-8 play a key role in systemic and chronic inflammatory conditions [42].

A chronic production of IL-1β by the respiratory epithelial cells of adult mice was related to lung inflammation, airway fibrosis, and mucus metaplasia [43]. IL-1β is a key mediator of neutrophilic inflammation in COPD [44], but it is also secreted by macrophages and CD4+ lymphocytes [92]. In COPD, IL-1β is associated with disease severity and induces mucus hypersecretion, contributing to airflow obstruction [92]. Mucus metaplasia and goblet cell hyperplasia are linked to chronic lung inflammation and BPD [43]. Tao et al. analyzed the inflammatory signature in the secreted sputum of preterm infants who received mechanical ventilation and found that IL-1β was elevated in the first week after oxygen therapy with no significant decrease until the fourth week [93]. An in vitro assay showed that hyperoxia significantly increased miR-34a expression, which is an IL-1β-positive regulator [93]. Furthermore, TNFAIP3 interacting protein 2 (TNIP2), an inhibitor of NFκB, is a direct target of miR34a and negatively regulates the production of IL-1β [93].

IL-6 is synthesized in the early stages of inflammation and plays a pivotal role in inducing and maintaining chronic inflammation [46]. Increasing evidence highlights its role in COPD: IL-6 levels are elevated in induced sputum of COPD patients and are inversely associated with lung function [45,94]. Two 3-year follow-up studies have reported that increased IL-6 concentrations in the serum of patients with COPD are related to higher mortality rates [47,95]. Augmented IL-6 levels in peripheral blood and BAL fluid were also detected in patients with BPD, together with increased plasma levels of oxidative stress markers [49,50]. Elevated IL-6 levels in the cord blood of premature infants were similarly associated with an increased risk of BPD [48]. An IL-6 knockout (−/−) murine experimental model showed that genetic IL-6 absence improved the inflammatory cascade associated with BPD development, reducing the risk of lung disease [96].

IL-8 is mainly secreted by stimulated macrophages and is a potent chemoattractant for neutrophils [57]. Higher levels of neutrophils and IL-8 have been found in the BAL fluid of infants who developed BPD compared to neonates who did not develop BPD [60]. A study on preterm infants described that increased cord blood IL-6 and IL-8 levels were related to a higher risk of developing moderate/severe BPD [58]. An increased risk of developing severe BPD at 36 weeks PGA was also associated with elevated IL-6 and IL-8 concentrations in the plasma of preterm infants [61]. Serum and sputum IL-8 concentrations are elevated in COPD patients, particularly in those who exhibit more severe disease or experience frequent exacerbations [51,59]. Three polymorphisms of the IL-8 gene (rs4073A, rs2227306C, and rs2227307T) were linked to a worse progression of COPD and a reduction in lung function over the years [52].

#### 3.2.2. TNF

TNF is a cytokine with several effects on various cellular types. It is considered a major regulator of inflammatory responses [53]. TNF is implicated in both BPD and COPD pathogenesis. A systematic review and meta-analysis published in 2019 reported that TNF levels are increased in COPD patients compared to healthy controls [54]. TNF increases lung inflammation and activates IL-8 and other pro-inflammatory mediators via the NFκB pathway in the airways [97]. Previous studies showed that hyperoxia stimulates TNF expression in murine lungs, facilitating the development of chronic lung disease [55,62]. Human studies reported increased TNF concentrations in tracheal aspirate samples of those newborns who subsequently developed BPD [56]. A recent systematic review showed an association between the rs1800629 polymorphism and susceptibility to BPD in preterm newborns [63].

#### 3.2.3. TGF-β

TGF-β is implicated in lung development. Overexpression of the TGF-β1 ligand in the lung altered late lung development, inducing changes in the alveolar stage [64,65]. Thus, TGF-β can be considered a negative regulator of alveolarization. In COPD, TGF-β facilitates the thickening of the alveolar wall by inducing fibrosis and predisposing to emphysema, contributing to cellular apoptosis [98]. In a study on a murine model, TGF-β stimulated pulmonary macrophages to produce augmented levels of matrix metalloproteinases (MMPs), which injured the alveoli, inducing emphysema [68]. Fibrosis is also detected in infants with BPD due to the overexpression of the TGF-β signaling pathway, which can be mediated by Smad3 signaling [69].

#### 3.2.4. VEGF

VEGF regulates vasculogenesis and angiogenesis in the human body. In the fetal lung, high-affinity receptors for VEGF are principally expressed in alveolar epithelial cells and myocytes, modulating the vascular endothelium [26]. Vascular growth is damaged in infants with BPD, and VEGF levels during the first week of life in newborns who subsequently develop BPD seem to be lower compared to neonates who do not develop BPD [26]. The inhibition of VEGF receptors in fetal lungs decreased endothelial nitric oxide synthase, leading to BPD [99]. In patients with COPD, VEGF levels were found to be increased in the bronchial, bronchiolar, and alveolar epithelium and in bronchiolar macrophages, as well as in airway and vascular smooth muscle cells in both the bronchiolar and alveolar regions [66,100]. VEGF receptors were also higher in patients with COPD compared with healthy subjects [100].

These biomarkers offer insights into the inflammatory and remodeling processes in COPD and BPD, potentially identifying BPD infants at higher risk of developing COPD. Further biomarkers, such as interferon (IFN)-γ, macrophage inflammatory protein (MIP)-1α, and monocyte chemoattractant protein (MCP)-1, have been previously discussed [67,70,71,92], but their role in the pathogenesis of BPD and COPD needs to be clarified in future studies.

### 3.3. Novel Biomarkers

In recent years, several novel molecules have been described as possible biomarkers in multiple lung diseases, including BPD and COPD.

#### 3.3.1. *PRMT7*

Epigenetic mechanisms can alter the regulation of lung alveologenesis in some conditions. Alveologenesis is the final step of lung development in which mature alveoli develop through a remodeling of primitive saccules [73]. Alterations during alveologenesis can have dramatic effects, leading to chronic lung diseases, such as BPD [74]. Alveologenesis involves the expansion of alveolar epithelium and alveolar myofibroblasts (AMYFs), which are a type of fibroblast located in the airways [72]. AMYF abnormalities have been described in both BPD and COPD [75]. Protein arginine methyltransferase 7 (*PRMT7*) is a type III enzyme responsible for monomethylation of arginine residues on both histone and nonhistone substrates [76]. *PRMT7* regulates AMYF proliferation and differentiation during lung alveologenesis [76]. In a study on mice, PRMT7 deficiency induced a reduction in AMYF proliferation and differentiation, abnormality in elastin deposition, and failure of alveolar septum formation [76]. AMYF proliferation and differentiation seem not to be altered by the overexpression of oncogene forkhead box M1 (Foxm1), a direct PRMT7 target. Foxm1 could be a potential target for intervention in pulmonary diseases such as BPD and COPD in future studies [76].

#### 3.3.2. Cathelicidin/LL-37

Cathelicidin/LL-37 is part of the human antimicrobial peptide family and plays a pivotal role in immune response [101]. Previous studies reported that decreased LL-37 levels could be a risk factor for developing severe COPD [102]. A human study showed that very preterm infants who displayed higher cord blood LL37 levels had a reduced risk of developing BPD [103]. IL-6 concentration was negatively correlated with LL37 and may be associated with the protective effect of LL37 on BPD [103].

#### 3.3.3. *CRISPLD2*

*CRISPLD2* (cysteine-rich secretory protein LCCL domain containing 2) is a glucocorticoid and developmentally regulated gene encoding a secreted mesenchymal protein in the lung and other organs [81]. This gene regulates airway branching morphogenesis and alveologenesis via mesenchymal–epithelial interactions [80]. In early murine embryogenesis, the absence of Crispld2 is lethal [80]. Heterozygous Crispld2 +/− mice develop a lung phenotype comparable to human BPD, including distal airspace enlargement, disruption of elastin, signs of neonatal lung inflammation (such as goblet cell hyperplasia), and elevated expression of pro-inflammatory mediators [80]. The suppression of endogenous *CRISPLD2* in adult lung fibroblasts led to higher expression of IL-8, IL-6, and C-C motif chemokine ligand 2 (CCL2), increasing the risk of developing COPD [104].

#### 3.3.4. *GDF15*

Growth differentiation factor 15 (*GDF15*) is a member of the TGF-β superfamily. Its expression increases in several pathological conditions and is associated with a senescence phenotype [82]. Cellular senescence plays an important role in many lung diseases, including BPD and COPD [77]. In patients with COPD, high levels of *GDF15* were associated with an increased yearly rate of exacerbations, higher mortality, and increased decline in lung function [105]. A study on preterm infants showed that *GDF15* could predict respiratory outcomes; in fact, increased levels were associated with longer mechanical ventilation need, prolonged respiratory support need, and prolonged length of hospital stay [79]. Future studies are needed to evaluate its potential role as a biomarker in both BPD and COPD.

### 3.4. Limitations and Clinical Relevance of Biomarkers

Although several inflammatory mediators are shared between BPD and COPD, many of the biomarkers discussed, including IL-6, IL-8, and TNF, lack disease specificity and are elevated in numerous inflammatory and infectious conditions. As such, these markers are unlikely to serve as stand-alone diagnostic or prognostic tools.

Importantly, key performance metrics required for clinical application—such as sensitivity, specificity, predictive values, and validated cutoff thresholds—have not been established for any of the biomarkers reviewed. Moreover, none has demonstrated long-term predictive validity for identifying infants with BPD who will develop COPD or COPD-like phenotypes later in life [51,52,53,59,61].

Emerging biomarkers, including *PRMT7*, cathelicidin/LL-37, *CRISPLD2*, and *GDF15*, are of interest due to their potential involvement in lung development, inflammation, and repair. However, current evidence is largely preliminary, often derived from animal models or small human cohorts, and its translational relevance remains uncertain [56].

Consequently, these biomarkers should currently be regarded as tools for mechanistic insight and hypothesis generation rather than clinically actionable markers.

### 3.5. Polygenic Risk Scores (PRS) and Lung Function Outcomes

Recent advancements in genomic medicine have shifted the focus from single-gene variants to polygenic risk scores (PRSs), which aggregate the effects of numerous common genetic variants across the genome to estimate an individual’s genetic liability to a specific trait or disease. In the context of respiratory outcomes, the application of PRS has shown significant predictive value. For instance, Nissen et al. demonstrated that a PRS derived from large-scale genome-wide association studies (GWAS) for chronic obstructive pulmonary disease (COPD) in adults is significantly associated with lung function trajectories in children born preterm [78]. Specifically, individuals with a higher polygenic burden for COPD exhibit lower FEV1 and FEV1/FVC ratios during childhood and adolescence, suggesting that genetic predisposition to adult respiratory disease may manifest early in life, particularly when combined with the physiological stress of prematurity [78]. Integrating PRS into clinical risk models could therefore enhance the identification of high-risk infants who may benefit from closer longitudinal monitoring and targeted preventive strategies.

## 4. Role of Smoking and Other Lifelong Risk Factors

Cigarette smoking remains the primary risk factor for COPD and plays a central role in disease initiation and progression [106]. In individuals with a history of early-life lung injury, such as BPD, smoking may act as a potent second hit, potentially amplifying pre-existing structural or functional vulnerabilities [2]. However, the impact of smoking may also overshadow any contribution from early developmental abnormalities.

In addition to smoking, a wide range of factors, including environmental and occupational exposures, recurrent respiratory infections, socioeconomic determinants, and genetic background, can independently influence lung function trajectories and COPD risk [78]. These factors underscore the multifactorial nature of COPD and highlight that multiple biological and environmental pathways can converge on similar clinical phenotypes [17].

Although genetic variants such as SERPINA1 and HHIP have been associated with both impaired lung development and COPD susceptibility, current evidence does not clearly demonstrate that these variants contribute to both BPD and COPD within the same individuals. Thus, genetic overlap should be interpreted as suggestive of shared susceptibility pathways rather than definitive proof of a unified disease mechanism.

Within this framework, BPD may represent one of several early-life conditions that contribute to reduced lung function reserve, increasing susceptibility to chronic obstructive lung disease later in life, particularly when combined with additional environmental or behavioral risk factors.

## 5. Disease Heterogeneity, Endotypes and Shared Sub-Phenotypes

Both bronchopulmonary dysplasia and chronic obstructive pulmonary disease are increasingly recognized as heterogeneous disorders encompassing multiple clinical phenotypes and biological endotypes [15]. In BPD, different sub-phenotypes have been described based on severity, lung structural abnormalities, degree of vascular involvement, and long-term respiratory outcomes [2]. Similarly, COPD comprises distinct phenotypes, including emphysema-predominant disease, chronic bronchitis, frequent exacerbators, and airway-dominant disease, which may be driven by different underlying inflammatory and molecular pathways [106].

Biomarkers represent a promising tool to dissect this heterogeneity by identifying endotypes characterized by shared pathophysiological mechanisms rather than solely clinical features. The overlap of inflammatory, oxidative, and vascular pathways observed in BPD and COPD suggests that a subset of individuals with BPD may develop a COPD-like endotype later in life, characterized by persistent airway inflammation, impaired lung repair, and accelerated lung function decline.

In this context, shared biomarkers such as pro-inflammatory cytokines, markers of endothelial dysfunction, and mediators of abnormal lung remodeling may help identify individuals at higher risk for specific long-term respiratory trajectories. Recognizing overlapping BPD–COPD endotypes could ultimately facilitate earlier risk stratification and support the development of targeted preventive and therapeutic strategies across the life course.

## 6. Conclusions

This narrative review highlights the significant biological overlap and shared susceptibilities between bronchopulmonary dysplasia (BPD) and chronic obstructive pulmonary disease (COPD). Despite emerging at opposite ends of the lifespan, these conditions are linked by common pathogenic pillars: persistent inflammation, vascular dysfunction, and impaired lung repair or remodeling. The evidence suggests that early-life lung injury in BPD may predispose individuals to COPD-like trajectories in adulthood, reflecting common biological responses to distinct insults rather than a simple disease continuum. Central to this interplay are key inflammatory mediators—such as IL-1β, IL-6, IL-8, TNF, and TGF-β—which drive chronic airway remodeling and fibrosis in both phenotypes. Furthermore, alterations in vascular-related factors like VEGF underscore a shared disruption in lung development and repair mechanisms. Emerging markers, including PRMT7, Cathelicidin/LL-37, CRISPLD2, and GDF15, are also gaining attention, offering the potential for deeper phenotypic characterization and a better understanding of disease-specific pathways. However, the clinical utility of these biomarkers is currently limited to the research setting. At present, they serve primarily as tools for hypothesis generation and long-term surveillance rather than for individualized risk prediction or targeted intervention. Since there are no established therapies specifically designed to prevent COPD in survivors of BPD, general preventive measures, such as smoking cessation and reduction in environmental exposures, remain the cornerstone of respiratory health across the life course. Future research must prioritize large-scale longitudinal studies to clarify the temporal relationship between early biomarker expression and adult respiratory outcomes. Ultimately, deciphering the molecular links between early and late chronic lung diseases will be essential to developing precise monitoring strategies and preventive approaches aimed at preserving lung function throughout the lifespan.

## Figures and Tables

**Table 1:** Common biomarkers in BPD and COPD.

Biomarkers	BPD	COPD
*Genetic biomarkers*		
*SERPINA1*	Pathway anomalies identified in BPD-PH via multiomic studies [37]. Low A1AT elastase inhibitory activity observed in severe BPD [38].	Variants linked to early-onset COPD [39]. *SERPINA1* hypomethylation (cg02181506 and cg24621042) associated with COPD, especially in smokers [40].
*NFκB*	Pathway anomalies identified in BPD-PH via multiomic studies [37].	Overexpression in COPD, particularly in smokers [41,42].
*VEGF*	Pathway anomalies identified in BPD-PH via multiomic studies [37].	Variants (rs833068G > A, rs833070T > C, and rs3024997G > A) linked to susceptibility [43].
miR-206	Downregulated [44].	Upregulated [44].
*HHIP*	Increased *HHIP* levels in extremely low-birth-weight infants who developed BPD [45]. *HHIP* variant rs13147758 (GG genotype) found to be protective against BPD onset [45].	*HHIP* variants associated with a higher risk of COPD [46].
*Inflammatory biomarkers*		
IL-1β	Elevated levels induced mucus metaplasia and goblet cell hyperplasia [47]. Increased in the first week and until the fourth week after oxygen therapy [48].	High levels contribute to neutrophilic inflammation, disease severity, and mucus hypersecretion [49,50].
IL-6	Increased in peripheral blood, BAL fluid, and cord blood [51,52,53]. Knockout models (genetic absence) show reduced BPD development [54] Linked to moderate/severe BPD [55,56].	Elevated in induced sputum and serum [57,58,59]. Inversely related to lung function [60]. Associated with higher mortality rates [61].
IL-8	Increased in BAL fluid and plasma of BPD infants [62]. Linked to moderate/severe BPD [55,56].	Elevated in severe COPD and in patients with frequent exacerbations [63,64]. Three polymorphisms (rs4073A, rs2227306C, and rs2227307T) worsen disease progression [65].
*TNF*	Higher levels in tracheal aspirate samples [66]. *Rs1800629* polymorphism linked to BPD susceptibility [67].	Increased in COPD patients [68]. Activates NFκB and other inflammatory pathways in the airways [69].
*TGF*	Overexpression disrupts late lung development and alveolarization [70,71]. Induces fibrosis via Smad3 signaling [72].	Contributes to alveolar wall thickening, fibrosis, cellular apoptosis, and emphysema [73]. Stimulates over-production of MMPs, linked to emphysema [74].
*VEGF*	Lower levels in the first week of life in BPD infants [31]. VEGF receptor inhibition disrupts endothelial nitric oxide signaling, leading to BPD [32]	Increased in bronchial, bronchiolar, and alveolar epithelium, and in vascular smooth muscle cells [75,76]. VEGF receptor expression is also elevated [75].
*Novel biomarkers*		
*PRMT7*	Deficiency reduces AMYF proliferation and differentiation, disrupting elastin deposition and alveolar septation [77].	Deficiency reduces AMYF proliferation and differentiation and disrupts elastin deposition [77].
*Cathelicidin*/*LL-37*	Higher cord blood levels reduce BPD risk [78].	Lower levels associated with severe COPD [79].
*CRISPLD2*	Heterozygous Crispld2 +/− mice develop BDP-like lung changes (distal airspace enlargement, elastin disruption, goblet cell hyperplasia, and inflammation) [80].	*CRISPLD2* suppression in lung fibroblasts increases IL-8, IL-6, and CCL2 levels, promoting COPD development [81].
*GDF15*	Higher levels associated with prolonged mechanical ventilation, respiratory support, and hospital stay [82].	Elevated levels linked to frequent exacerbations, higher mortality, and accelerated lung function decline [79].

BPD = bronchopulmonary dysplasia; COPD = chronic obstructive pulmonary disease; NFκB = nuclear factor kappa-B; *VEGF* = vascular endothelial growth factor; miR-206 = microRNA-206; *HHIP* = hedgehog interacting protein; IL = interleukin; *TNF* = tumor necrosis factor; *TGF* = transforming growth factor-β; AMYFs = alveolar myofibroblasts; *PRMT7* = protein arginine methyltransferase 7; *CRISPLD2* = cysteine-rich secretory protein LCCL domain containing 2; GDF15 = growth differentiation factor 15; BPD-PH = pulmonary hypertension associated with BPD; *A1AT* = alpha-1-antitrypsin; BAL = bronchoalveolar lavage; MMPs = matrix metalloproteinases; CCL2 = C-C motif chemokine ligand 2.

## Data Availability

No new data were created or analyzed in this study. Data sharing is not applicable to this article.

## Abbreviations

The following abbreviations are used in this manuscript:

A1AT	Alpha-1-antitrypsin
AMYFs	Alveolar myofibroblasts
BAL	Bronchoalveolar lavage
BPD	Bronchopulmonary dysplasia
BPD-PH	Pulmonary hypertension associated with bronchopulmonary dysplasia
CCL2	C-C motif chemokine ligand 2
COPD	Chronic obstructive pulmonary disease
*CRISPLD2*	Cysteine-rich secretory protein LCCL domain containing 2
ERK	Extracellular signal-regulated kinase
FEV1	Forced expiratory volume in one second
Foxm1	Forkhead box M1
*GDF15*	Growth differentiation factor 15
GWAS	Genome-wide association studies
HIF-1	Hypoxia-induced factor 1
HHIP	Hedgehog interacting protein
IL	Interleukin
LL-37	Cathelicidin antimicrobial peptide
MCP-1	Monocyte chemoattractant protein-1
MIP-1α	Macrophage inflammatory protein-1α
MMPs	Matrix metalloproteinases
miR-206	MicroRNA-206
NFκB	Nuclear factor kappa-B
PGA	Postmenstrual gestational age
PRMT7	Protein arginine methyltransferase 7
PRS	Polygenic Risk Scores
SNPs	Single-nucleotide polymorphisms
SERPINA1	Serpin family A member 1
TGF-β	Transforming growth factor-beta
TNF	Tumor necrosis factor
TNIP2	TNFAIP3 interacting protein 2
VEGF	Vascular endothelial growth factor
VEGFR	Vascular endothelial growth factor receptor
